# Robotic distal gastrectomy plus spleen-preserving distal pancreatectomy: optimal resection for simultaneous gastric cancer and intraductal papillary mucinous neoplasm of the pancreatic body

**DOI:** 10.1186/s40792-024-01831-y

**Published:** 2024-02-09

**Authors:** Sunao Ito, Hiroyuki Sagawa, Kohei Fujita, Masaki Saito, Shinnosuke Harata, Shunsuke Hayakawa, Kenta Saito, Tatsuya Tanaka, Mamoru Morimoto, Ryo Ogawa, Hiroki Takahashi, Yoichi Matsuo, Shuji Takiguchi

**Affiliations:** Department of Gastroenterological Surgery, Graduate School of Medical Sciences, Nagoya City University, 1 Kawasumi, Mizuho-Cho, Mizuho-Ku, Nagoya, Aichi 467-8601 Japan

**Keywords:** Gastrectomy, Indocyanine green imaging, Pancreatectomy, Robotic surgical procedures

## Abstract

**Background:**

Organ-preserving surgery has recently gained increasing attention. However, performing the surgery for duplicated gastric and distal pancreatic tumors is difficult because of procedural complexity and concerns of remnant gastric necrosis. We present the first case of simultaneous robotic distal gastrectomy plus spleen-preserving distal pancreatectomy in a patient with overlapping gastric cancer and intraductal papillary mucinous neoplasm.

**Case presentation:**

A 78-year-old man was diagnosed with gastric cancer in the middle stomach and intraductal papillary mucinous neoplasm of the pancreatic body. Radical cure surgery was performed using the da Vinci Xi robotic system. Conventional distal gastrectomy was initially completed using near-infrared ray guidance when transecting the stomach. After dividing the pancreas, the parenchyma of the distal pancreas was detached from the splenic artery and vein; multiple branches from these splenic vessels were dissected. Indocyanine green imaging confirmed sufficient blood flow in the splenic vessels and perfusion of the remnant stomach. Ultimately, gastrointestinal reconstruction was performed, and the postoperative course was uneventful.

**Conclusions:**

The robotic distal gastrectomy plus spleen-preserving distal pancreatectomy procedure was safely performed. Compared to the total gastrectomy plus distal pancreatectomy with splenectomy procedure, this technique may improve the quality of dietary life, reduce weight loss, and prevent complications associated with splenectomy.

## Background

Conventional surgical resection procedures for gastrointestinal and pancreatic tumors are based on resection of the primary organ and lymph node dissection. Furthermore, the appropriate extent of resection has become more specific over time. Organ-preserving surgery has recently gained increasing attention, including stomach-preserving surgeries such as proximal gastrectomy and pylorus-preserving gastrectomy for gastric cancer (GC) [1] and spleen-preserving distal pancreatectomy (SPDP) for distal pancreatic tumors [2].

However, organ-preserving surgery for duplicated gastric and distal pancreatic tumors is challenging because of procedural complexity and concerns of remnant gastric necrosis.

Herein, we present a case of simultaneous distal gastrectomy (DG) and SPDP in a patient with overlapping GC and intraductal papillary mucinous neoplasm (IPMN) of the pancreatic body. To the best of our knowledge, this is the first case report of a robotic approach.

## Case presentation

**Fig. 1:**
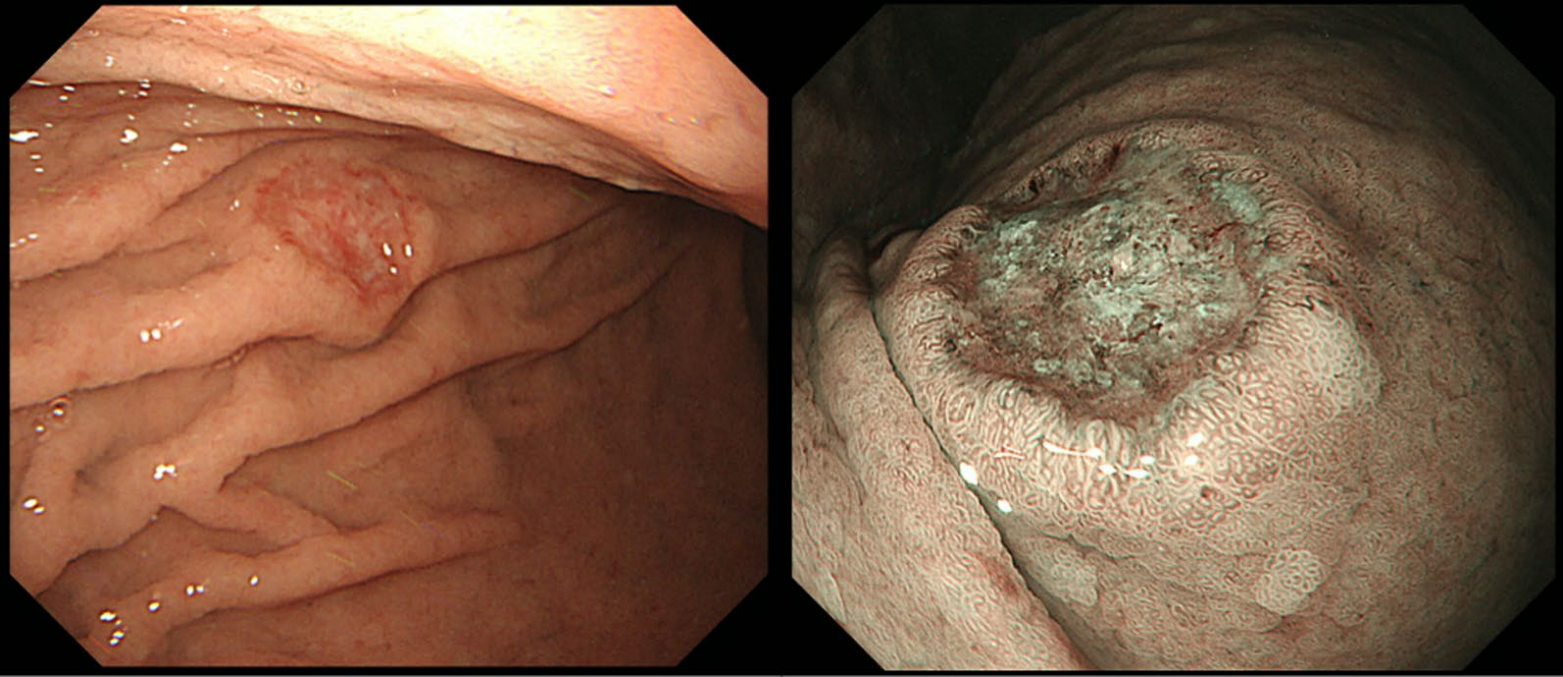
A 10-mm type 0–III lesion was observed on the greater curvature of the middle stomach. The tumor was diagnosed as a highly differentiated tubular adenocarcinoma

**Fig. 2:**
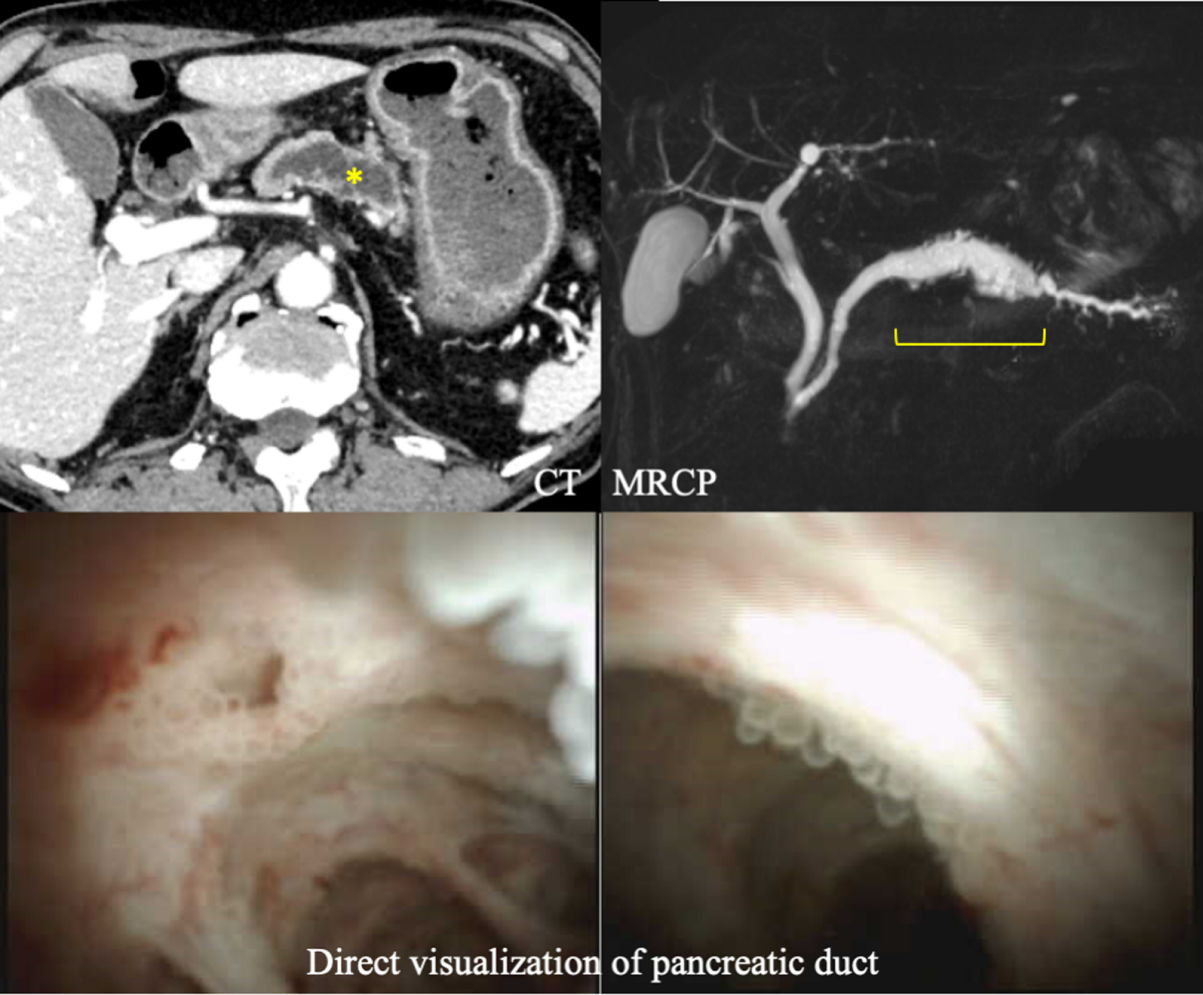
CT and MRCP showed that the main pancreatic duct was dilated to a diameter of 10 mm along a length of 50 mm. No mural nodules or enlarged peripancreatic lymph nodes were observed. The lower pictures show direct visualization of the papillary epithelium in the main pancreatic duct. CT, computed tomography; MRCP, magnetic resonance cholangiopancreatography

**Fig. 3:**
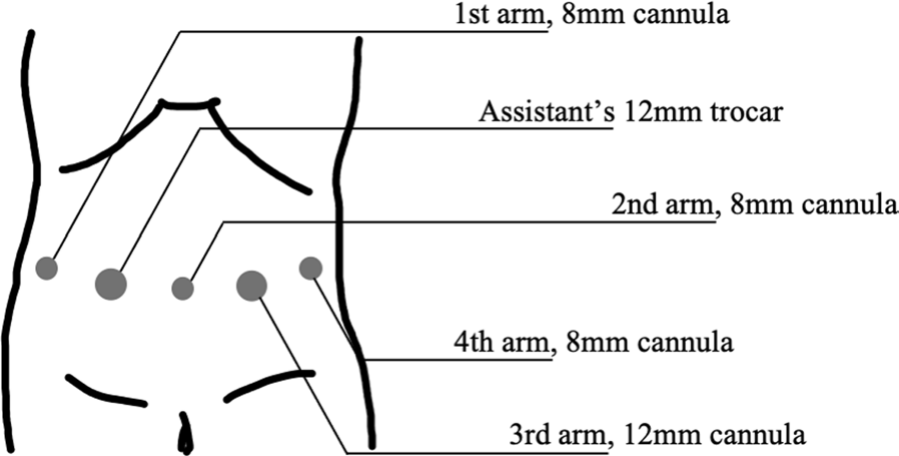
Port placement for the surgery

**Fig. 4:**
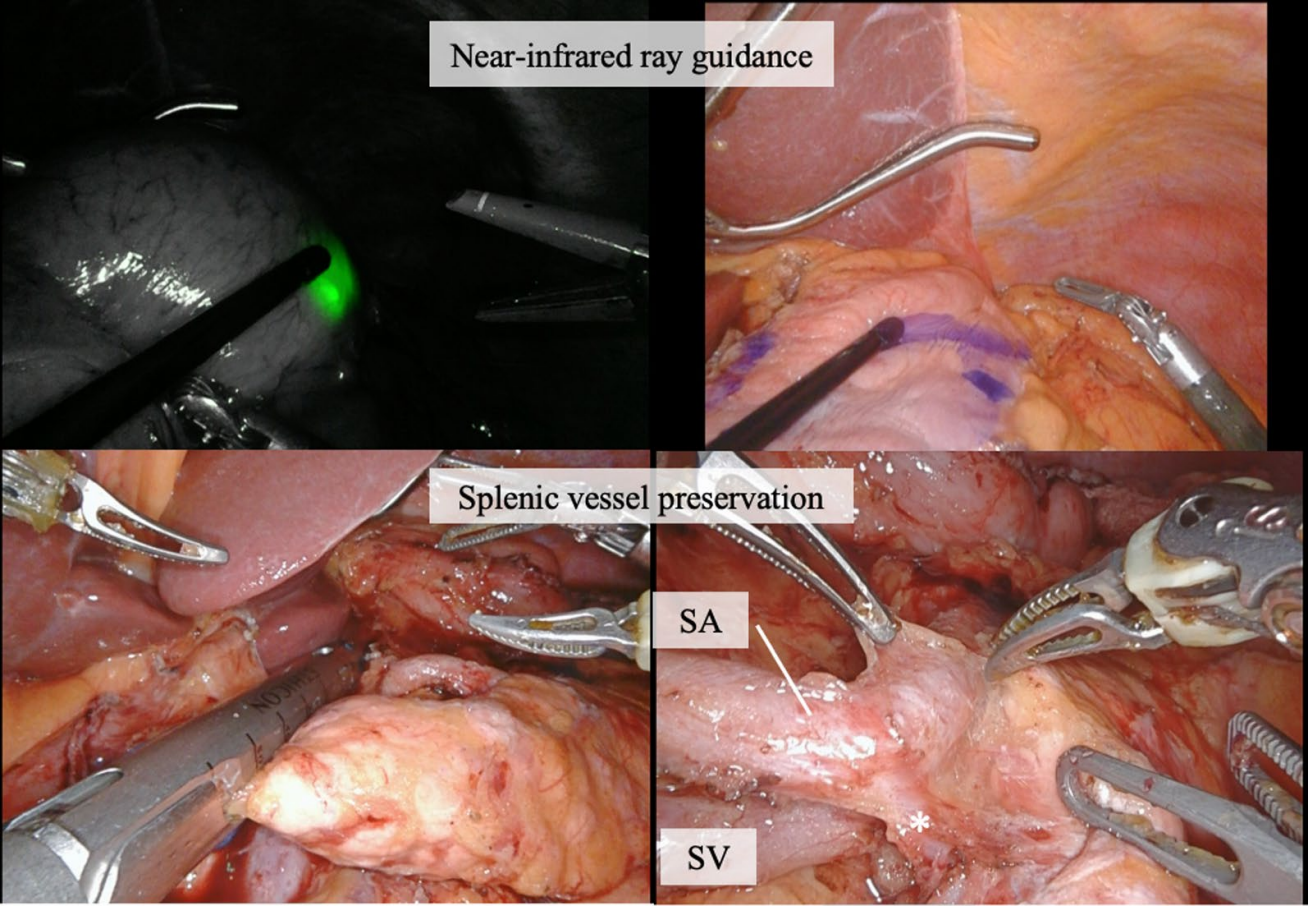
An adequate cutoff position was determined using near-infrared ray guidance. Distal pancreatectomy was performed with preservation of the splenic artery and vein. *Artery branching to the pancreas. *SA* splenic artery; *SV* splenic vein

The patient was a 78-year-old man. Systemic examination prior to tongue cancer (TC) treatment identified GC and IPMN of the pancreas. A 10 mm GC was present in the middle stomach and was clinically diagnosed as T1N0 (Fig. 1) based on the 8th edition of the TNM Classification of Malignant Tumors. The IPMN was located on the pancreatic body, was of the main pancreatic duct type, and had a maximum diameter of 10 mm. No mural nodules or enlarged peripancreatic lymph nodes were observed (Fig. 2), and biopsy of the papillary epithelium revealed adenoma (Fig. 3). TC was clinically determined as T1N2b (Fig. 4), the most advanced of the three tumors; hence, partial resection of the right tongue and cervical lymph node dissection were performed first. Two months after the patient’s condition improved, the patient underwent simultaneous resection of the GC and IPMN. Robotic surgery using the da Vinci Xi (Intuitive Surgical, Sunnyvale, CA, USA) was performed. The port placement for the surgery was the same as that in conventional robotic DG (Fig. 3), and no changes were made for pancreatic resection. First, gastrectomy was performed. Initially, lymphadenectomy and vascular division of the greater curvature were performed, and the duodenum was dissected. Subsequently, the lesser curvature was approached from the right to the left side, the right and left gastric artery/vein were dissected, and suprapancreatic lymphadenectomy was performed. The perisplenic arterial lymph nodes were dissected to facilitate subsequent pancreatectomy.

**Fig. 5:**
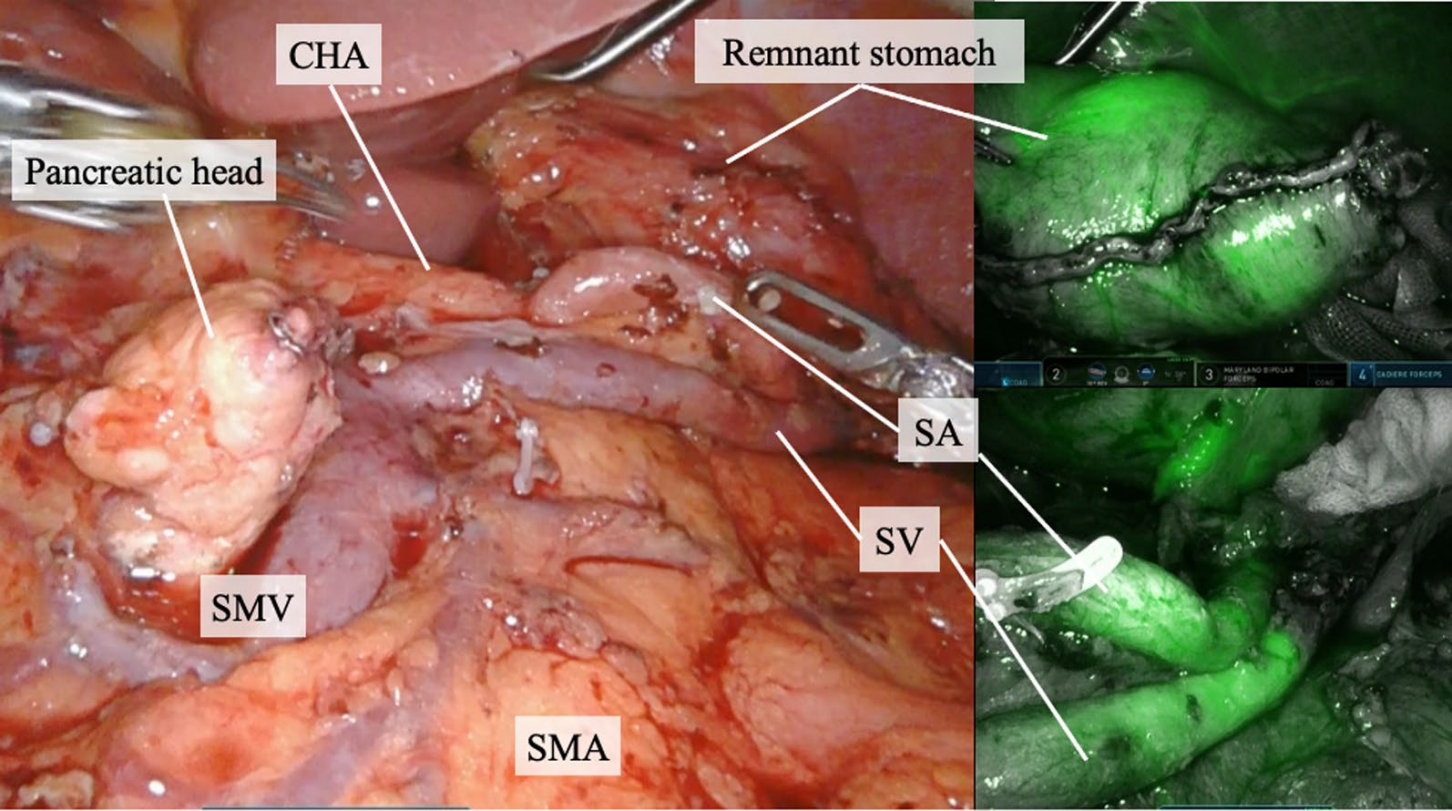
The surgical field after resection. Indocyanine green imaging shows adequate blood flow of the splenic vessels and remnant stomach. *CHA* common hepatic artery; *SA* splenic artery; *SMA* superior mesenteric artery, *SMV* superior mesenteric vein; *SV* splenic vein

The near-infrared ray-guided method [3] was applied to identify the localization of the small gastric tumor (Fig. 5). The stomach was transected along the markings using the near-infrared ray-guided method, and the specimen was removed from the abdomen to confirm complete tumor resection.

**Fig. 6:**
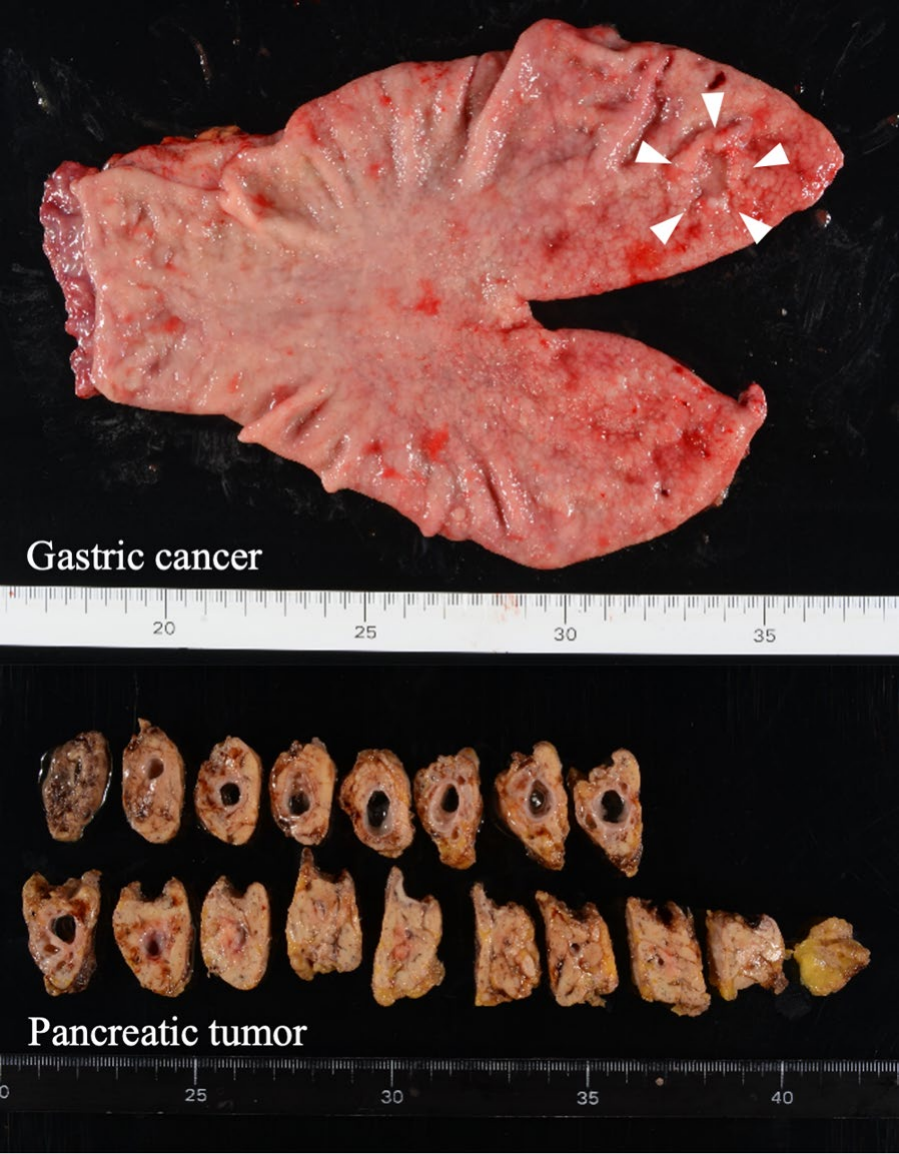
Resected specimens. Arrowheads indicate the location of the gastric cancer

Following gastrectomy, pancreatic resection was performed. Tunneling was conducted between the pancreas and superior mesenteric vein, and the pancreas was divided using an automated suturing device. The parenchyma of the distal pancreas was detached from the splenic artery (SA) and vein using Maryland bipolar (Intuitive Surgical, Sunnyvale, CA, USA). Furthermore, multiple branches of these splenic vessels were dissected: the branches of the splenic vein were dissected with Vessel Sealer Extend (Intuitive Surgical, Sunnyvale, CA, USA) and the branches of the SA were clipped on the central side before dissection with Vessel Sealer Extend. After complete resection of the distal pancreas, indocyanine green was administered intravenously (0.05 mg/kg), and sufficient blood flow in the splenic vessels and perfusion of the remnant stomach were confirmed 25 s after injection using the Firefly mode (Fig. 6). Finally, Billroth II anastomosis was performed. The operative time was 551 min, the volume of blood loss was 216 ml, and the postoperative course was uneventful. The pathological diagnosis was pT1bN0 pStage IA for gastric cancer and intraductal papillary mucinous adenoma for pancreatic tumor, and no postoperative chemotherapy was administered.

The patient was 155 cm tall and weighed 48.2 kg before surgery. The patient experienced the greatest amount of weight loss during the first postoperative month, reaching 46.5 kg (3.5% loss), and regained his original weight of 48.5 kg in the second postoperative month. The patient maintained the same weight for more than 2 years. However, 2.5 years postoperatively, TC recurred and chemotherapy was initiated.

## Discussion

Robotic DG plus SPDP was safely performed in a patient with overlapping GC and IPMN of the pancreatic body. This procedure allows organ preservation without compromising treatment effectiveness. Preservation of the upper stomach and spleen may lead to long-term prognosis by maintaining the dietary quality of life and avoiding late complications.

Regarding radical cure and the optimal extent of resection, standard DG and lymphatic dissection are required for middle GC; however, simultaneous distal pancreatectomy (DP) potentially decreases blood flow to the remnant stomach due to SA ligation, which may lead to remnant stomach necrosis. Therefore, total gastrectomy plus DP is generally preferred as a safe procedure. However, surgery for IPMN does not require lymphadenectomy; therefore, dissection of the SA is unnecessary. While SPDP is a complicated and time-consuming procedure, it was performed for optimal resection of IPMN, consequently preserving blood flow in the SA system and enabling standard DG for gastric cancer. However, this procedure has been rarely reported [4, 5]. The present study is the first report of a robotic approach for DG plus SPDP. Central pancreatectomy can be considered instead of SPDP as a less invasive procedure if a reliable method for preventing pancreatic fistula has been established.

The advantages of the robotic process include the ability of the surgical robot to provide high-resolution three-dimensional images and enable precise surgical manipulation using tremor-filtered articulated forceps. As a result, the robotic approach reduces complications of laparoscopic gastrectomy [6]. It may also be suitable for procedures that require meticulous care, such as vessel-sparing surgery. The success rate of laparoscopic SPDP is reported to be 76.6% [7], and further improvements are expected when using a robotic approach. It should be noted that port placement is important, and if the port position of the 3rd arm is too close to the SA, manipulation will be difficult. Therefore, shifting the port position of the 3rd arm a few centimeters toward the caudal side may improve maneuverability and allow a more stable operation. Furthermore, the da Vinci Xi is equipped with Firefly mode that enables near-infrared ray guidance and real-time blood flow evaluation [3, 8], allowing easy assessment of the resection line of the stomach and blood flow in the splenic vessels, respectively, thereby contributing to improved surgical safety.

The preservation of the stomach and spleen significantly influences postoperative outcomes. First, stomach preservation may reduce weight loss and improve the quality of dietary life [9, 10], potentially contributing to a favorable long-term prognosis. In the present case, postoperative weight loss was limited to 3.5%, and the patient maintained his recovered original weight for > 2 years, starting 2 months postoperatively. Second, spleen preservation prevents complications such as coagulopathy and overwhelming post-splenectomy infection (OPSI). Although treatments such as pharmacotherapy for coagulopathy and vaccine therapy for OPSI are available, avoiding the risks associated with complications is preferable.

## Conclusions

Robotic DG plus SPDP was safely performed in a patient with overlapping GC and IPMN of the pancreatic body. While technically demanding, this surgical method achieves radical tumor cure and organ preservation, making it a highly valuable treatment option. Compared to the total gastrectomy plus DP with splenectomy procedure, robotic DG plus SPDP potentially improves the patient’s quality of dietary life, reduces weight loss, and prevents complications due to splenectomy, thereby contributing to a favorable long-term prognosis.

## Data Availability

Not applicable to this paper as no datasets were generated during this study.

## Abbreviations

GC: Gastric cancer
SPDP: Spleen-preserving distal pancreatectomy
DG: Distal gastrectomy
IPMN: Intraductal papillary mucinous neoplasm
TC: Tongue cancer
SA: Splenic artery
OPSI: Overwhelming post-splenectomy infection
DP: Distal pancreatectomy
